# Cardiac Implantable Electronic Device Placement in the Era of Transcatheter Tricuspid Replacement: Approaches and Challenges

**DOI:** 10.19102/icrm.2025.16112

**Published:** 2025-11-15

**Authors:** Robert D. Schaller, Mikhael F. El-Chami

**Affiliations:** 1 Section of Cardiac Electrophysiology, Cardiovascular Division, Department of Medicine, Hospital of the University of Pennsylvania, Philadelphia, PA, USA; 2 Cardiology Division, Emory University School of Medicine, Atlanta, GA, USA

**Keywords:** Implantable cardioverter-defibrillator, lead entrapment, lead jailing, leadless pacing, pacemaker, transcatheter tricuspid valve replacement

## Abstract

Transcatheter tricuspid valve (TV) interventions (TTVIs), which include transcatheter TV replacement (TTVR) and transcatheter tricuspid edge-to-edge repair (T-TEER), represent a natural evolution in percutaneous valve therapy. However, TTVIs face distinct challenges, chief among them being the frequent presence of transvenous cardiac implantable electronic device (CIED) leads crossing the TV. This review investigates contemporary CIED strategies that eliminate the need for transvenous leads crossing the TV, thereby facilitating safer and more durable integration of device therapy with emerging TTVI technologies.

## Introduction

Transcatheter tricuspid valve (TV) interventions (TTVIs) represent a natural evolution in percutaneous valve therapy,^1^ inspired by the transformative success of transcatheter aortic valve replacement (TAVR).^2,3^ These procedures include transcatheter TV replacement (TTVR) and transcatheter tricuspid edge-to-edge repair (T-TEER) with the EVOQUE (Edwards Lifesciences, Irvine, CA, USA) and the TriClip (Abbott, Chicago, IL, USA) systems, which are currently the only commercially available options for each approach in the United States, respectively. Unlike TAVR, which must navigate the complexities of left-sided lesions and transient obstruction of arterial flow, TTVIs face distinct challenges, chief among them being the frequent presence, either current or anticipated, of transvenous cardiac implantable electronic device (CIED) leads crossing the TV.^4^ While T-TEER systems are designed to navigate around these pre-existing leads during deployment, TTVR poses more significant concerns regarding long-term lead function and the feasibility of future percutaneous transvenous lead extraction.

**Figure 1:**
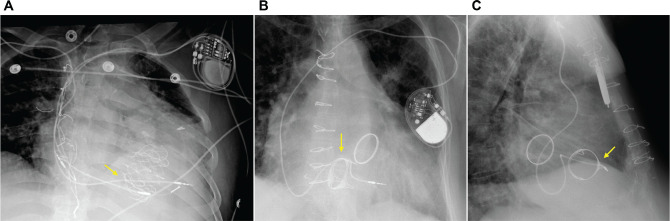
**A:** Frontal chest radiograph of a patient with two right ventricular leads that have been entrapped by a transcatheter tricuspid valve replacement (arrow). Posterior–anterior **(B)** and lateral **(C)** radiographs of a right ventricular lead that has been entrapped by a surgical tricuspid valve replacement (arrows).

Despite the widespread use of right ventricular (RV) leads, they have been largely overlooked in clinical trials evaluating TTVI, with rates of transvenous RV leads in clinical studies involving TTVR or T-TEER ranging from 3%–36%.^5^ In the context of TTVR, lead entrapment or “jailing” can have important clinical consequences. These include an increased risk of lead dysfunction, which, although there are limited data, has been reported in up to 10% of cases over short-term follow-up^6^ **(Figure 1A)**. More concerning is the potential loss of percutaneous lead extractability in the event of infection. This may force reliance on suboptimal alternatives such as prolonged antibiotic therapy, local pocket debridement, intentional laceration of leads with retraction into the chest, non-traditional percutaneous lead extraction with unpredictable risks, or high-risk surgical extraction in patients already deemed poor surgical candidates. These strategies, previously encountered with leads entrapped by surgical TVRs, increase morbidity and mortality and represent a deviation from the standard of care **(Figures 1B and 1C)**.^7^

New-onset conduction disturbances requiring permanent pacing have also been reported in 2%–15% of patients following TTVR, driven by the proximity of the conduction system to the septal TV annulus and influenced by device-specific factors such as septal anchoring and radial forces.^8^ Patients with pre-existing conduction disease are particularly susceptible, with most new abnormalities occurring within the first week, underscoring the need for early monitoring, particularly in high-risk patients.

In response to these challenges, the use of multidisciplinary “heart teams”—including electrophysiologists with expertise in lead management—has been recommended to assess the risks and benefits of lead extraction versus entrapment on a case-by-case basis. These teams can also help determine the most appropriate modality for permanent pacing, if needed, following TTVR.^5^ When lead extraction is pursued, alternative CIED strategies should be considered, including deferring reimplantation in patients who no longer require device therapy. Similarly, for patients undergoing initial CIED implantation who may require TTVR in the future, proactive planning is essential. This review aims to serve as a comprehensive reference for contemporary CIED strategies that eliminate the need for transvenous leads crossing the TV, thereby facilitating safer and more durable integration of device therapy with emerging TTVI technologies.

## Single-chamber atrial pacemakers

**Figure 2:**
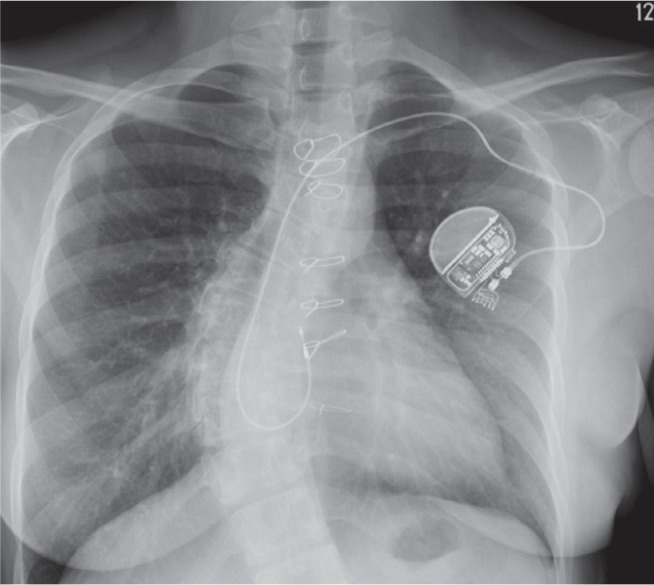
Frontal chest radiograph of a patient with a single-chamber atrial pacemaker.

Patients with sinus node dysfunction (SND) do not inherently require ventricular pacing **(Figure 2)**. Nevertheless, traditional permanent pacemaker (PPM) systems im-planted in the United States for this indication have often included an RV lead to account for the potential, albeit low, risk of high-grade atrioventricular (AV) block, and to avoid the need for re-entering the device pocket in the future.^9^ The addition of an RV lead, however, may have unintended consequences beyond its impact on future TV interventions. Notably, CIED leads are implicated in 10%–15% of cases of worsening tricuspid regurgitation (TR), either by mechanical interference or pacing-induced dyssynchrony.^10,11^

Patients with SND who receive an RV lead despite not requiring ventricular pacing often rely on ventricular pacing minimization algorithms to reduce the risk of pacing-induced cardiomyopathy, even subacutely.^12,13^ While these algorithms effectively limit unnecessary ventricular pacing, observational data have not shown a significant advantage over standard DDD programming in reducing the incidence of persistent atrial fibrillation (AF), all-cause hospitalization, or mortality.^14^ Similarly, the randomized DANPACE trial found no significant difference in all-cause mortality between AAIR and DDDR pacing; however, AAIR pacing was associated with a greater incidence of paroxysmal AF and a twofold increased risk of PPM reoperation.^15^ These trade-offs highlight the need for shared decision-making when selecting pacing strategies in patients with isolated SND, particularly when the P–R interval is within normal limits and the QRS is narrow. Should ventricular pacing be required in the future, alternative strategies such as lead placement through a TTVR or within the coronary sinus (CS) system remain viable options.

## His-bundle pacing

**Figure 3:**
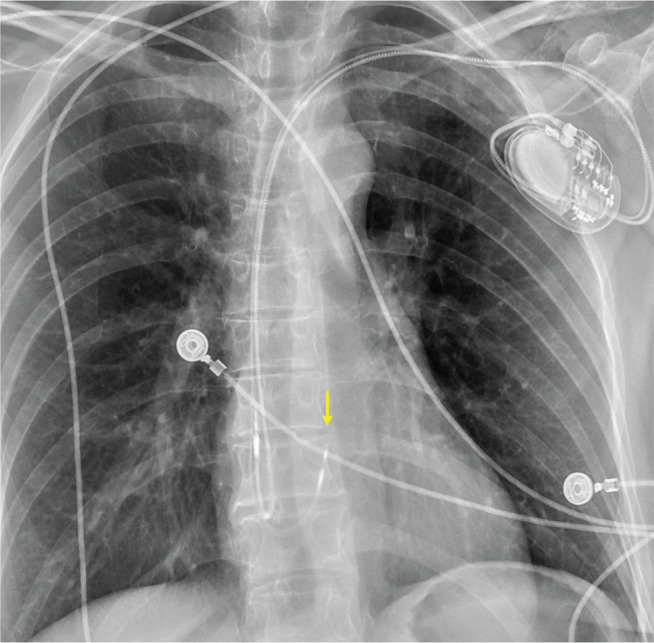
Frontal chest radiograph of a patient with a dual-chamber pacemaker that includes a His-bundle pacing lead (arrow).

Prior to the rapid adoption of left bundle branch area pacing (LBBAP), His-bundle pacing (HBP) emerged as the first novel conduction system pacing (CSP) technique, gaining significant attention through a grassroots movement that reinvigorated the pacing field **(Figure 3)**.^16^ A distinct advantage of HBP in the setting of TTVR is the ability to position the lead on the atrial side of the TV annulus.^17^ As with surgical TVR, the right atrial segment of the His bundle remains anatomically intact, and successful HBP from the right atrium has been demonstrated in this context.^18,19^ Moreover, successful HBP implantation has been reported even after TTVR.^20^ This makes HBP an attractive pacing strategy both before and after TTVR, as it enables the conduction system to capture without transvalvular lead placement.

However, post-implant feasibility may be limited by technical challenges, and the impact of TTVR on pacing thresholds and lead performance in patients with pre-existing HBP systems remains poorly understood. Enthusiasm for HBP has also diminished with the rise of LBBAP, which, unlike HBP, necessitates crossing the TV.

## Ventricular pacing leads exclusively within the coronary sinus system

### Permanent pacemakers

Cardiac resynchronization therapy (CRT) using biventricular (BiV) devices with leads positioned in the CS is a cornerstone therapy for patients with dyssynchrony-induced cardiomyopathy. Traditionally, a CS lead functions in conjunction with an RV lead to ensure appropriate timing or to provide redundancy. The incidence of CS lead dislodgement or malfunction within the first year post-implantation remains low, reported at approximately 1.4% in contemporary systems.^21^ While single CS leads have been used in select cases, these are typically bipolar leads, which offer fewer pacing configurations compared to the more widely adopted quadripolar leads.^22,23^ However, this approach has been shown to be feasible and safe, albeit with longer fluoroscopy times as compared to traditional RV leads.^24^

**Figure 4:**
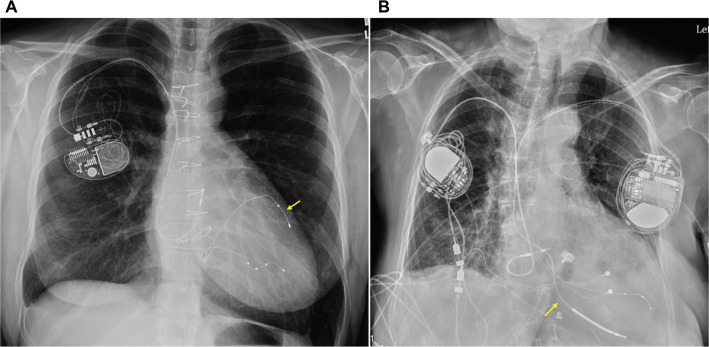
**A:** Frontal chest radiograph of a patient with a biventricular permanent pacemaker with two ventricular leads in the coronary sinus system. A bipolar lead (arrow) is in the anterior interventricular vein and a quadripolar lead is in a posterolateral branch. **B:** Frontal chest radiograph of a biventricular implantable cardioverter-defibrillator system with an implantable cardioverter-defibrillator lead in the middle cardiac vein (arrow) connected to a left-sided generator. Of note, there is a non-functional right-sided permanent pacemaker system that includes an epicardial ventricular lead.

More commonly, two leads are placed within the CS—a bipolar lead positioned deep within the anterior interventricular vein or middle cardiac vein (MCV), and a quadripolar lead in a lateral vein **(Figure 4A)**.^25,26^ This approach provides multiple pacing vectors and ensures redundancy, particularly in PPM-dependent patients.

### Implantable cardioverter-defibrillators

Traditional implantable cardioverter-defibrillators (ICDs) typically rely on a transvenous RV lead, often positioned near the ventricular apex to optimize defibrillation thresholds (DFTs). While non-transvenous ICD systems are an option for select patients, many still benefit from intravascular leads due to the need for pacing capabilities. Numerous case reports have described successful placement of ICD leads within the CS system, including both the main CS and its distal branches. Given the importance of proximity to the ventricular apex for effective defibrillation, shocking coils are generally preferred in distal CS branches. In cases where high DFTs necessitate optimization of the defibrillation vector or a reduction in impedance, a second coil may be placed in the main body of the CS.^27,28^ Additional coil locations also reported include the innominate vein,^29^ persistent left superior vena cava,^30^ subcutaneous tissue,^31^ and azygous vein.^32^

Placement of a single ICD lead outside of the RV is uncommon; however, a recent report described five successful ICD implantations within the CS system, predominantly in the MCV **(Figure 4B)**.^33^ Due to the anatomical proximity to the ventricular apex, DFTs are typically acceptable. However, pacing and sensing via the epicardial surface through coronary veins can be suboptimal, occasionally necessitating an additional pace-sense lead in a separate region of the CS.

### Leadless permanent pacemakers

**Figure 5:**
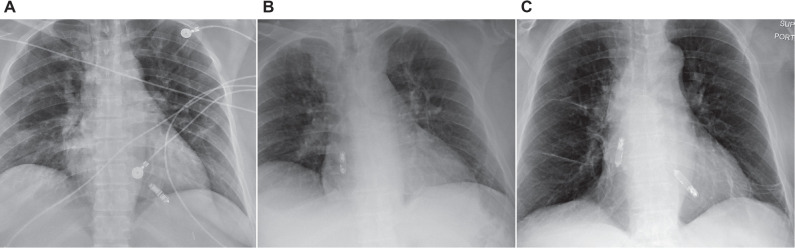
Frontal chest radiograph of leadless permanent pacemaker (PPM) options. **A:** A single-chamber leadless PPM in the right ventricle. **B:** A single-chamber leadless PPM in the right atrium. **C:** A dual-chamber PPM in the right atrium and ventricle.

Leadless PPMs filled a critical gap in the pacing landscape after being first introduced a decade ago.^34^ Patients without suitable upper-extremity vascular access or those at high risk for CIED-related infections now had an alternative to traditional transvenous systems. Initially limited to single-chamber devices implanted in the RV (Micra™ [Medtronic, Minneapolis, MN, USA] or AVEIR VR [Abbott]) **(Figure 5A)**, early indications were restricted. However, the development of accelerometer-based atrial sensing and enhanced algorithms (Micra™ AV; Medtronic) significantly improved AV synchrony, expanding applicability to those with adequate sinus rates.^35^ More recently, a fully dual-chamber leadless PPM became commercially available (AVEIR DR; Abbott),^36^ using a novel implant-to-implant communication modality, in which sub-threshold electrical pulses are transmitted between devices to maintain AV synchrony. While the atrial version of this device (AVEIR AR; Abbott) can be used independently **(Figure 5B)**, early data on dual-chamber safety, the degree of AV synchrony, and long-term electrical performance have been favorable **(Figure 5C)**.^37^

**Figure 6:**
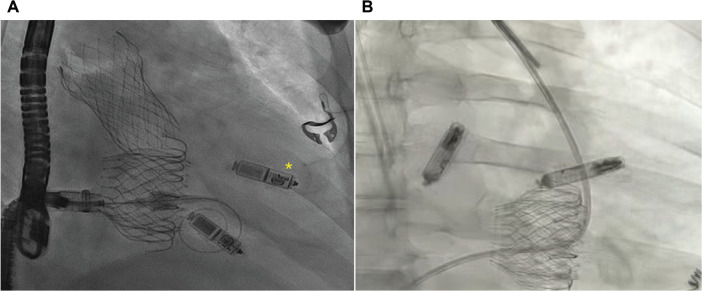
Frontal chest radiographs of patients with pre-existing leadless pacemakers undergoing transcatheter tricuspid valve replacement (TTVR). **A:** Deployment of a TTVR in a patient with two Micra™ devices. The mid-septal device (asterisk) is the more recent implant. **B:** A TTVR implanted in a patient with an AVEIR DR. The ventricular AVEIR is implanted in the mid-high septum, and there is clear interaction between the valve and the leadless device.

Although the delivery sheaths for these devices are large (>25 F), implantation has been shown to be safe even in challenging scenarios such as in the setting of TVR or TTVR.^38–40^ This is facilitated by the use of advanced deflectable delivery systems in combination with orthogonal fluoroscopy, intracardiac echocardiography, and contrast injection in both chambers. Importantly, when considering leadless PPM implantation either before or after TTVR, careful attention must be paid to device location and overall length. The Micra™ and AVEIR VR leadless pacemakers measure 25.9 mm and 38 mm in length, respectively—a nearly 1-cm difference that may increase the risk of interaction with the prosthetic valve, particularly when the device is positioned at a mid- to high-septal location **(Figure 6)**.

While current leadless pacing systems have demonstrated excellent AV synchrony, they remain limited by the inability to deliver CSP. A first-in-human acute feasibility study of the AVEIR CSP (Abbott)—featuring an extended pacing and sensing electrode with a distal helix designed to penetrate the interventricular septum and enable LBBAP—was recently reported.^41^ Successful septal implantation was achieved in 10 of 12 patients via the right internal jugular vein. Acceptable pacing parameters were observed, with LBBAP, left ventricular septal pacing, or deep septal pacing attained in 8 of 10 successful cases. The ability to reliably deliver leadless CSP would represent a landmark advancement in the evolution of cardiac pacing and an ideal pacing solution for patients after TTVI.

## Non-transvenous implantable cardioverter-defibrillators

### Subcutaneous implantable cardioverter-defibrillator

**Figure 7:**
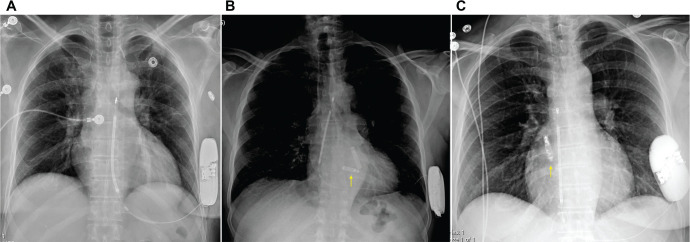
**A:** Frontal chest radiograph of a subcutaneous implantable cardioverter-defibrillator (S-ICD). **B:** An S-ICD system that includes a leadless pacemaker in the right ventricle (arrow) with the ability to deliver anti-tachycardia pacing. Note there is also an implantable cardiac rhythm therapy device. **C:** An S-ICD system and a separate atrial AVEIR device.

The completely subcutaneous ICD (S-ICD) was a major addition to the CIED landscape providing patients without venous access, a high risk of infection, or a preference to avoid transvenous leads with an option for life-saving therapy **(Figure 7A)**.^42^ The S-ICD, which includes a generator along the left midaxillary line and a suprasternal shocking lead, has been shown to be non-inferior to traditional ICDs with fewer complications and lower rates of infection, albeit with a higher rate of inappropriate shocks.^43–45^ The S-ICD has been used in a vast array of patient populations with improving implantation techniques^46^ and can be safely used before or after median sternotomy.^47^

One limitation of the S-ICD is its inability to deliver anti-tachycardia pacing (ATP), a reliable and painless therapy for monomorphic ventricular tachycardia. The recently reported modular pacing–defibrillator system, consisting of an S-ICD paired with a leadless RV PPM (EMPOWER; Boston Scientific, Marlborough, MA, USA) **(Figure 7B)**, demonstrated 98.8% successful wireless communication between devices and a 61.3% successful ATP termination rate.^48^ Although not yet commercially available in the United States, this technology has the potential to expand the indications for the S-ICD to patients with a need for ATP.

In select cases—such as limited venous access or DFT concerns—the S-ICD may be combined with a transvenous or leadless PPM **(Figure 7C)**. When pairing separate devices, it is critical to confirm that the paced morphology is appropriately sensed by the S-ICD.^49^ An alternative implantation strategy that has been described involves the simultaneous placement of a surgical dual-chamber PPM with epicardial leads on the left atrial and left ventricular surfaces at the time of S-ICD implantation, using the same surgical incision. This approach has been shown to be both feasible and effective, offering a single-procedure solution that avoids leads crossing the TV.^50^

### Extravascular implantable cardioverter-defibrillator

**Figure 8:**
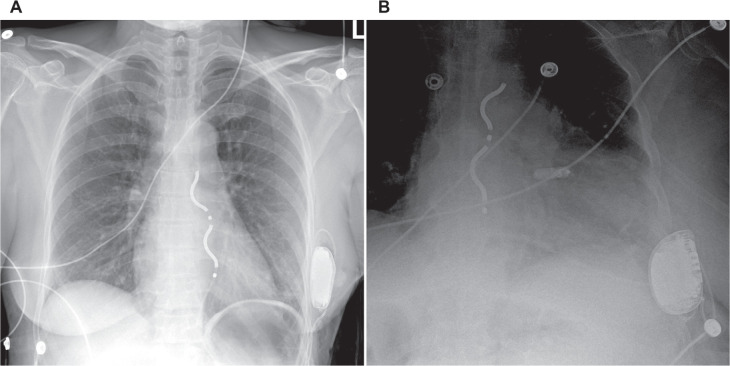
**A:** Frontal chest radiograph of an extravascular implantable cardioverter-defibrillator (EV-ICD) that includes a substernal lead. **B:** An EV-ICD with a separate leadless right ventricular pacemaker.

The extravascular ICD (EV-ICD) is a novel ICD that similarly features a generator positioned along the left midaxillary line but uses a substernal shocking lead **(Figure 8A)**. The proximity of the lead to the heart enables delivery of ATP, which was successfully delivered 50.8% of the time in the pivotal trial.^51^ Despite the unique lead location, no major intraprocedural complications were reported; however, 29 patients experienced more inappropriate shocks than anticipated during follow-up, primarily due to atrial oversensing. While this remains a concern, optimization of sensing during implantation and incorporation of a P-wave oversensing discriminator—now included in the current device iteration—have shown promising improvements.^52^ Various combinations of leadless devices have also been described, including use of the EV-ICD in combination with a leadless PPM **(Figure 8B)**.^53^

## Leads placed through a transcatheter tricuspid valve replacement

**Figure 9:**
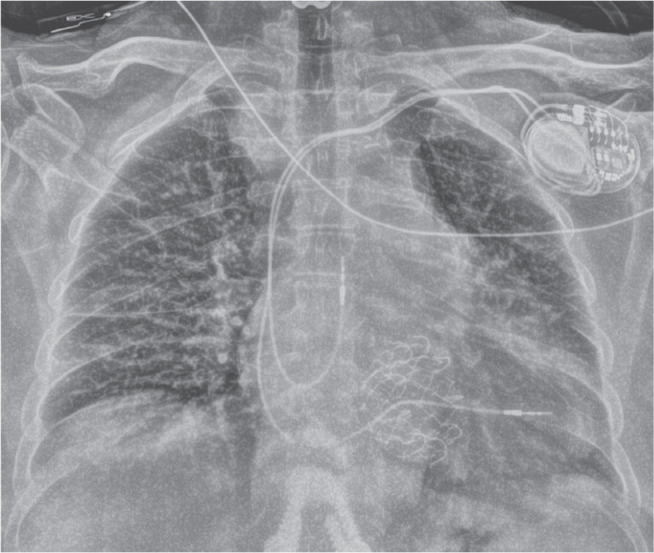
Frontal chest radiograph of a pacing lead that has been placed through a transcatheter tricuspid valve replacement.

Data on the long-term performance of TTVRs with transvalvular CIED leads remain limited, though insights can be drawn from the surgical TVR experience. While lead placement through a prosthetic TV is generally discouraged due to risks of lead-related TR—including leaflet impingement, adherence, perforation, entanglement, or fibrotic fusion—existing data do not consistently demonstrate worsened clinical outcomes. In fact, studies suggest that transvalvular leads are not clearly associated with an increased incidence of TR in this population.^54,55^ However, the high incidence of conduction disturbances following surgical TVR, coupled with the potential risks of lead passage through prosthetic valves, has led many to favor prophylactic strategies such as epicardial PPM implantation at the time of surgery. While TTVR avoids the morbidity associated with surgical TVR, it precludes epicardial lead placement and complicates pacing strategies, making conventional RV pacing, including LBBAP, a more attractive option. Although leads have been successfully placed through TTVRs^56^ **(Figure 9)**, the long-term implications remain unknown. Given concerns about future valve-in-valve procedures and the lack of robust data, placing leads across a TTVR should likely not be considered a first-line strategy.

## Surgical permanent pacemaker systems

**Figure 10:**
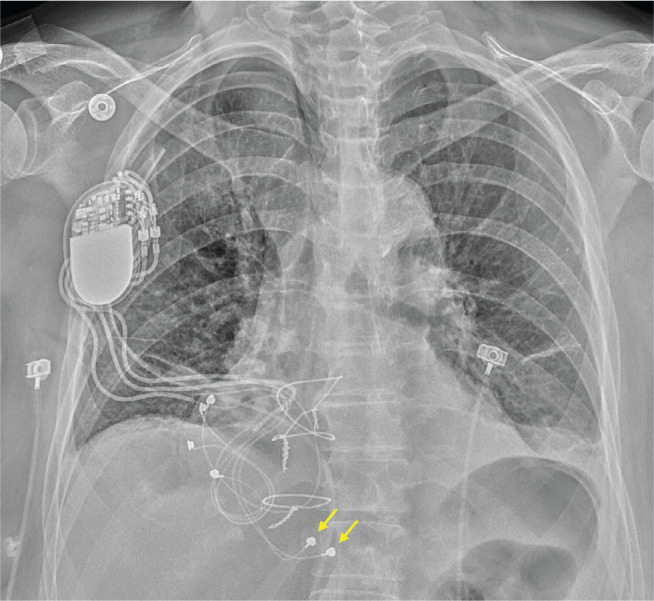
Frontal chest radiograph of a patient with a completely epicardial dual-chamber pacemaker system with leads overlying the right atrium and right ventricle (arrows).

Surgical epicardial PPM systems have been performed for decades using well-established techniques and modern, reliable leads that offer acceptable longevity, even in patients with prior cardiac surgery or complex congenital heart disease.^57–59^ These systems can be implanted via a subxiphoid approach, thoracotomy, median sternotomy, or minimally invasive video-assisted thoracoscopic surgery^60^ **(Figure 10)**. Multiple epicardial leads can be placed on the atria and ventricles, enabling configurations supporting BiV pacing or redundancy, and the generator can be positioned in the abdomen or chest wall depending on anatomical and surgical considerations.

Most data on the long-term outcomes of epicardial PPM systems come from pediatric populations. However, these systems are also frequently used in adults—particularly during concomitant cardiac surgery, in patients without venous access, those with lead-related venous swelling, and in cases requiring CRT after failed percutaneous attempts.^61,62^ The surgical morbidity associated with epicardial lead placement varies by approach and must be carefully weighed against the benefit of avoiding transvenous leads on a patient-specific basis. To facilitate future revisions, the generator should be tunneled to the chest wall, and lead redundancy should be incorporated to account for potential lead failure over time.

## Surgical implantable cardioverter-defibrillator systems

Completely surgical epicardial ICD systems are inherently more complex than PPM systems due to the need for one or more shocking coils on the epicardial surface. While early-generation ICDs were exclusively surgical and relied on epicardial defibrillation patches,^63^ issues related to patch performance and the rapid adoption of transvenous systems led to a decline in innovation in this area.^64^ As a result, defibrillation patches are no longer manufactured.

**Figure 11:**
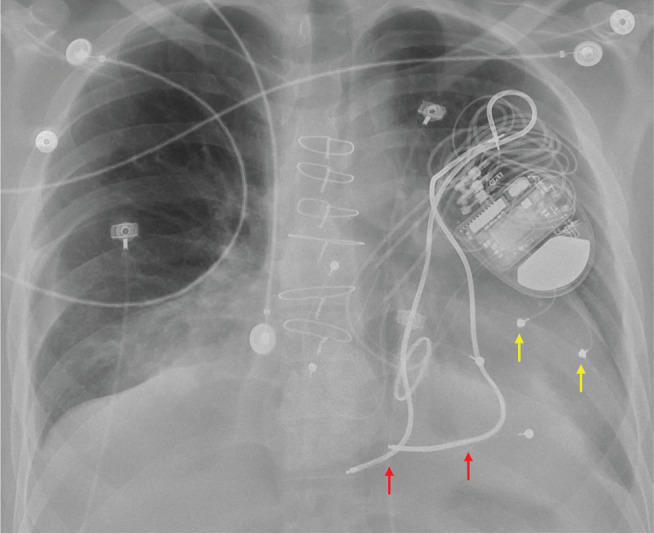
Frontal chest radiograph of a patient with a completely biventricular implantable cardioverter-defibrillator system. Note the pacing leads (yellow arrows) as well as shocking coils (red arrows).

Contemporary epicardial ICD systems typically use one or more standard transvenous ICD leads that are surgically sutured to the heart. Various surgical approaches have been employed—including subxiphoid, thoracotomy, and sternotomy techniques—although these systems generally require more extensive exposure than epicardial PPM implantation **(Figure 11)**. Because these leads cannot be actively fixated to the heart via a helix for appropriate sensing, separate pace-sense leads must be implanted alongside the shocking coils. Although experience remains limited, small case series have demonstrated successful implantation of epicardial ICDs, including CRT defibrillator systems, without evidence of epicardial infection, coronary artery compression, constrictive pericarditis, or coil erosion into intrathoracic structures.^65^

## Conclusion

The era of TTVIs is well underway and will continue to evolve rapidly. As this field advances, it is imperative for the electrophysiology community to proactively adapt pacing strategies to minimize future complications, including lead entrapment, malfunction, and the implications of infection. Emerging real-world data on the consequences of jailed leads will likely reinforce the urgency for forward-looking solutions. Future approaches will increasingly rely on the expanding technologies of leadless pacing, non-transvenous ICD systems as well as alternative lead placement techniques driven by clinical necessity. These innovations will evolve in parallel with advancing TTVI platforms, including transcatheter bicaval valve systems.^66,67^ Close collaboration with interventional colleagues through local and regional heart teams, along with transparent data sharing across the electrophysiology and cardiovascular communities, will be critical to inform clinical decision-making and drive continued innovation. The landscape of cardiac pacing is poised for irreversible transformation, and it is incumbent upon us to evolve with it.
